# Development of a Prostatic Urethral Cyst After Rezum Causing Persistent Lower Urinary Tract Symptoms

**DOI:** 10.7759/cureus.49969

**Published:** 2023-12-05

**Authors:** Nojoud A AlAmri, Khadijah Eid, Mohammed AlShehri

**Affiliations:** 1 Urology, King Abdullah Bin Abdulaziz University Hospital, Riyadh, SAU; 2 Urology, Princess Nourah Bint Abdulrahman University, Riyadh, SAU

**Keywords:** prostatic hyperplasia, prostatic urethra, urethral cyst, complications, luts, rezum

## Abstract

Water vapor thermal therapy (WVTT) is a new modality. There are just a few short periods of outcome surveillance following Rezum, with a maximum term of five years. The majority of side effects are irritative lower urinary tract symptoms caused by endoscopic manipulation and typically last a short time. Herein, we present a novel case of a 71-year-old male patient with a Rezum-induced prostatic urethral cystic lesion. Five months following Rezum, an ultrasound was performed to evaluate persistent lower urinary tract symptoms (LUTS) revealed the lesion. The patient preferred conservative management rather than cystoscopy and ablation of prostatic urethral cyst.

## Introduction

Rezum is a technique that utilizes water vapor thermal energy at the prostate. It decreases prostate volume by inducing tissue necrosis with moderate levels of thermal energy, lowering the risk of treatment-related morbidity [1]. It has an excellent result compared with traditional thermal therapies such as conductive transurethral needle ablation of the prostate (TUNA) and transurethral microwave thermotherapy (TUMT) [1].

New minimally invasive surgical therapies (MISTs) have emerged. Rezum belongs to MISTs, demonstrating the ability to respect and preserve sexual function while providing safety and minimal morbidity [2]. The Rezum procedure has several advantages, including prolonged symptomatic relief in high-risk patients who are unfit for Transurethral resection of the prostate (TURP) and no de novo cases of ejaculatory dysfunction. Compared with standard medical therapies, it offers significantly better outcomes for quality of life (QoL), International Prostate Symptom Score (IPSS), and prostate volume [1].

Another key advantage is that it can be conducted as a day-case operation in an outpatient setting and under local anesthesia because it is a minimally invasive procedure. Rezum, unlike many other BPH treatment modalities, can target and treat the prostatic median lobe [1]. We herein report a case of the development of a prostatic urethral cyst after Rezum, causing persistent Lower urinary tract symptoms.

## Case presentation

A 71-year-old male patient presented with a history of diabetes, hyperlipidemia, and prostatic enlargement and was on alfuzosin. Additionally, the patient complained of urgency and frequency, which did not improve after using Solifenacin. He did not have any history of previous urine retention, hematuria, hesitancy, or nocturia. Uroflowmetry showed the total volume of voided urine was 360 milliliter (mL), the total flow time was 21 sec (second), and the maximum flow rate (Qmax) was 37 mL/sec. The prostate-specific antigen (PSA) was 0.4 nanograms per milliliter (ng/mL). Urine tests showed no leukocytes, nitrates, or bacteria. The urine culture was negative.

Ultrasound of the kidney and bladder revealed that the bladder wall was slightly irregular; the pre-void bladder capacity was 262 mL, and the post-void urine residual was 67 mL. The prostate volume measured 21.5 mL, and both kidneys showed normal size and echogenicity. Cystoscopy showed mildly obstructive prostatic lobes, an enlarged median lobe, a high bladder neck, and mildly trabeculated bladder walls. The patient underwent Rezum and tolerated the procedure without complications. Rezum was delivered to the prostate in three injections, one in each lobe. Subsequently, the Foley catheter was removed after five days, and the pre-operative complaints of the patient had improved during the post-operative follow-up.

**Figure 1:**
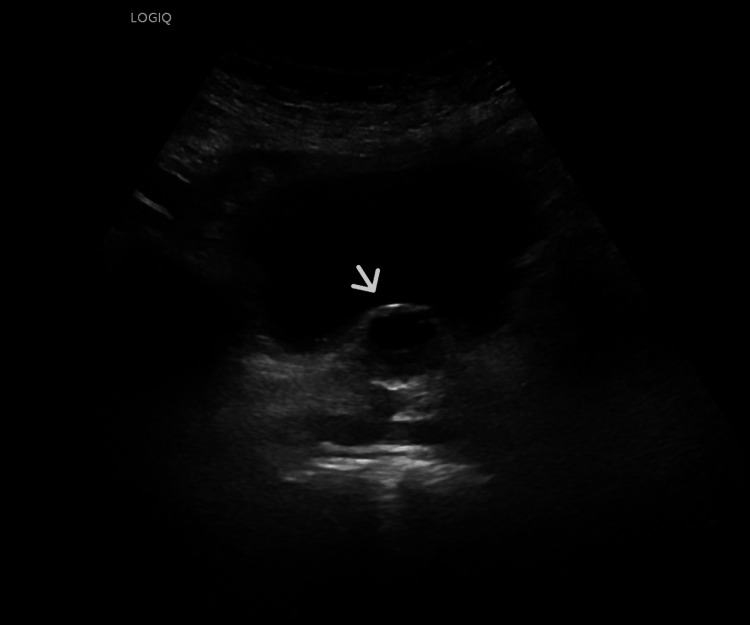
US bladder showed prostatic central anechoic mass measures 2.2* 1.6 ml

**Figure 2:**
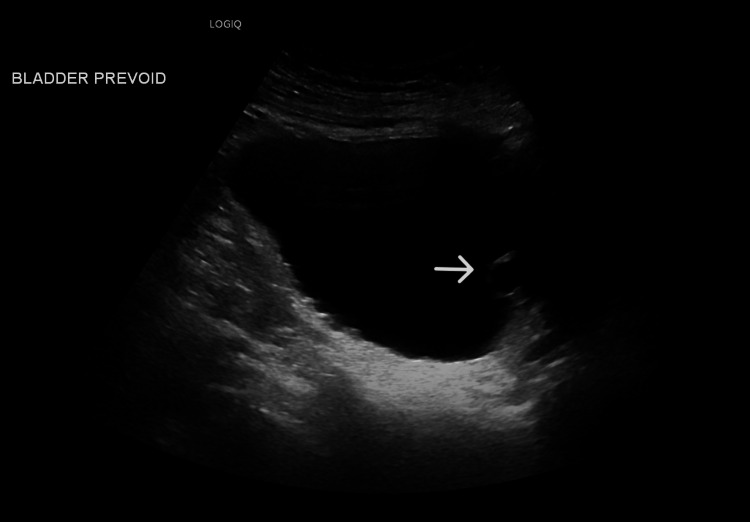
Sagittal view US bladder showed a prostatic anechoic mass

Five months after the Rezum procedure, the patient presented with dysuria and urgency. The status of desire, erection, and ejaculation in comparison to pre-operative status was stable. Uroflowmetry showed that the total volume of the urination was 337 mL, the total flow time was 35 sec, and the Qmax was 39 mL/sec. Ultrasound of the bladder indicated that the prostate volume measured 24 mL, demonstrating prostatic central anechoic mass measuring 2.2*1.6 mL (Figure 1, 2). Though the patient was offered a choice between conservative management or cystoscopy and prostatic urethral cyst ablation, the patient preferred conservative treatment.

## Discussion

Rezum is a new modality, with a few studies describing the procedure, the possible complications, and long-term outcomes. Only one study conducted post-Rezum outcome surveillance, and it had relatively short follow-up periods, with a maximum duration of five years [3]. Although most of the reported side effects were due to endoscopic instrumentation and epithelial shedding and lasted for a short period [4], three months after thermal therapy, there was a significant improvement lasting five years. IPSS and the Benign Prostatic Hyperplasia Impact Index decreased by 48%, while the QOL and Qmax increased by 45% and 44%, respectively. There were no reports of procedure-related sexual dysfunction or prolonged new-onset erectile dysfunction. Furthermore, the surgical retreatment rate was 4.4% [3].

Patients with prostate size measuring less than 30 g and urinary retention experienced considerable relief from LUTS, with 83% of urinary retention patients being catheter-free in a median of 14 days [5]. In our case, the prostate size was 21.5g, and the symptoms improved until Five months after surgery [5]. The published studies showed a significant median percent improvement in IPSS, QOL, and Qmax at 3 months with 51%, 51%, and 66%, respectively [5].

However, after Rezum, most adverse effects were temporary and non-serious, occurring in 0-76% of patients (median 29%) [5]. There has been one reported encapsulated hematoma after Rezum therapy, in which the patient went to the emergency department with hematuria and clot retention two months after Rezum [6].

Here, we discussed with the patient the options of conservative management versus cystoscopy and prostatic urethral cyst ablation after ultrasound findings of a prostatic urethral cyst. However, the patient opted for conservative treatment. Though all published studies stated LUTS as the predominant complaint following the Rezum procedure, a recent study revealed that after Rezum, persistent LUTS caused three patients to develop anatomical changes that necessitated retreatment [7].

The main limitation of Rezum is that it does not produce tissues that can be used to collect samples for histopathology; therefore, it cannot detect incidental cases of prostate cancer. Additionally, patients with urinary retention or high prostatic size of more than 80g may be ineligible for Rezum.

## Conclusions

The development of prostatic urethral cysts after the Rezum operation is a rare complication. Although LUTS is the most common symptom, only a few cases underwent an ultrasound of the bladder as part of the investigation of persistent LUTS after Rezum surgery. Hence, we recommend including a bladder ultrasound during the three-month follow-up after Rezum. Additionally, It may help understand the anatomical changes, define the consequences, and indicate the need for surgical intervention.
